# Temporal tendencies and spatial patterns of human sporotrichosis in Rio de Janeiro State, Brazil, 2007 to 2023

**DOI:** 10.1590/S1678-9946202668002

**Published:** 2026-01-30

**Authors:** Rafael Ramalho Cunha-e-Silva, Sandro Javier Bedoya-Pacheco, Alex de Oliveira Vasconcelos, Mônica de Avelar Figueiredo Mafra Magalhães, Cristina Maria Giordano Dias, Dayvison Francis Saraiva Freitas, Sandro Antonio Pereira, Anna Barreto Fernandes Figueiredo, Juliana Gonçalves dos Reis, Paula Maria Pereira de Almeida, Cláudia Lima Campos Alzuguir, Liliane de Fátima Antonio Oliveira, Maria Inês Fernandes Pimentel

**Affiliations:** 1 Instituto Nacional de Infectologia Evandro Chagas Laboratório de Pesquisa Clínica e Vigilância em Leishmanioses Fundação Oswaldo Cruz Rio de Janeiro Brazil Fundação Oswaldo Cruz, Instituto Nacional de Infectologia Evandro Chagas, Laboratório de Pesquisa Clínica e Vigilância em Leishmanioses, Rio de Janeiro, Rio de Janeiro, Brazil; 2 Escola Nacional de Saúde Pública Sérgio Arouca Fundação Oswaldo Cruz Departamento de Epidemiologia e Métodos Quantitativos em Saúde/DEMQS Rio de Janeiro Brazil Fundação Oswaldo Cruz, Escola Nacional de Saúde Pública Sérgio Arouca, Departamento de Epidemiologia e Métodos Quantitativos em Saúde/DEMQS, Rio de Janeiro, Rio de Janeiro, Brazil; 3 Instituto de Comunicação e Informação Científica e Tecnológica em Saúde Fundação Oswaldo Cruz Rio de Janeiro Brazil Fundação Oswaldo Cruz, Instituto de Comunicação e Informação Científica e Tecnológica em Saúde, Rio de Janeiro, Rio de Janeiro, Brazil; 4 Secretaria de Estado de Saúde do Rio de Janeiro Rio de Janeiro Brazil Secretaria de Estado de Saúde do Rio de Janeiro, Rio de Janeiro, Rio de Janeiro, Brazil; 5 Universidade Federal do Rio de Janeiro Faculdade de Medicina Rio de Janeiro Brazil Universidade Federal do Rio de Janeiro, Faculdade de Medicina, Rio de Janeiro, Rio de Janeiro, Brazil

**Keywords:** Sporotrichosis, Zoonosis, Epidemiology, Spatial analysis, Neglected infectious disease

## Abstract

Cat-transmitted sporotrichosis is one of the fastest spreading zoonosis in Rio de Janeiro State, Brazil. A retrospective study was conducted with analysis of incidence of human sporotrichosis complemented with spatial methodologies. Data from case reported to the Notifiable Diseases Information System (SINAN) from 2007 to 2023 were studied. Incidence, demographic variables, temporal, and spatial dynamics of this endemic disease were investigated. During 2007–2023, 15,401 cases of sporotrichosis were reported. Most cases (64.4%) occurred with women. The annual incidence from 2007 to 2023 was 5.6 cases per 100,000 inhabitants. The incidence in 2016–2023 was 2.3 times higher than in 2007–2015. No significant differences were found regarding age between the two periods, but there were differences regarding gender: with a higher proportion of women in 2007–2015. The endemic is heterogenous with variations in time and space. Spatial analysis showed statistically significant clusters spread throughout Rio de Janeiro, in the periods of low incidence (2007–2015) and high incidence (2016–2023). In the period of high incidence, clusters were more numerous and had a greater range. In conclusion, the incidence levels and the proportion of affected territories increased over time. This study may contribute to understanding the dynamics of the endemic disease in the Rio de Janeiro State and guide control actions in places where they are most needed.

## INTRODUCTION

Sporotrichosis is one of the most important zoonoses in the Rio de Janeiro State, currently considered the region with the highest incidence in Brazil and in the world. This is a disease caused by species of fungi of the genus *Sporothrix*. The classic mode of transmission occurs via contaminated organic material (saprobiosis), with traumatic implantation leading to the development of cutaneous and subcutaneous lesions. This form of transmission originated the name "rose gardener's disease" by which sporotrichosis is also known^1^, due to the possibility of infection via trauma with thorns. Throughout most endemic areas of the world, sporotrichosis is associated with a saprobiotic origin, generally affecting rural workers and people who handle plants due to their professional or leisure activities^2-8^. The involvement of domesticated animals or rodents has been recognized since the beginning of the 20^th^ century^9,10^, and cases of human sporotrichosis acquired from cats were reported decades later^11^. This zoonotic mode of transmission from infected animals is currently the predominant one in the Rio de Janeiro State.

*Sporothrix* genus comprises dozens of species, most of which are non-pathogenic, with the so-called pathogenic clade including *S. schenckii, S. brasiliensis*, *S. globosa*, and *S. luriei*^12-14^. Virulence varies greatly among the different pathogenic species, with *S. brasiliensis* being considered a highly virulent species^12,13,15^.

The disease already existed sporadically in the Rio de Janeiro State in the last century. However, from 1998 onwards, an increase in the number of human cases was identified and associated with bites or scratches from infected cats^16^. These patients lived mainly in the capital of the Rio de Janeiro State and in some neighboring cities^17^. Fungi isolated from the nails of some cats involved in the transmission to humans were *Sporothrix spp.*, which reinforced the epidemiological link between cats and humans.^16^ Currently, it is established that the zoonotic endemic in Rio de Janeiro is caused by *S. brasiliensis*^18,19^, with the occurrence of cases by *S. schenckii* sensu stricto being much rarer^18^. Additionally, *S. brasiliensis* remains in the environment for years, further enabling the transmission to animals and humans^20^.

The increase in the number of human and feline cases led to the establishment of sporotrichosis as a notifiable disease in the Rio de Janeiro State in 2013^21^, which only became a Brazilian national policy in 2025, after the spread of the zoonosis to other states. In the current scenario, it is essential to conduct epidemiological analyses to understand the impact on the territories and the dynamics of the disease. This study aims to identify territorial patterns of sporotrichosis over time in different cities of Rio de Janeiro, based on indicators and spatial analyses.

## MATERIALS AND METHODS

### Study design

A retrospective study was conducted with analysis of incidence of human sporotrichosis complemented with spatial methodologies. This study was approved by the Research Ethics Committee of the Evandro Chagas National Institute of Infectious Diseases, Oswaldo Cruz Foundation (CAAE Nº 35591220.3.0000.5262).

### Study area

The study area was the Rio de Janeiro State (area: 43,753 km^2^), located in the Southeast region of Brazil, with a population of approximately 17 million people distributed across 92 cities.

### Study population and data source

Data from patients diagnosed with sporotrichosis reported to the Notifiable Diseases Information System (SINAN) in the Rio de Janeiro State, from 2007 to 2023, were studied. The reported cases included in this study were classified as "confirmed" in the SINAN system. Confirmation was based on laboratory and/or clinical-epidemiological criteria. Information included skin color (race), gender, age, residence address (city, neighborhood, street), probable city of infection (self-referred), work-related infection or not, method of diagnosis, disease progression (outcome). Population data were obtained from population estimates by the Brazilian Institute of Geography and Statistics (IBGE).

### Procedures

1. Temporal analysis of cases: annual time series interval.
2. Incidence rates: annual incidence rates in the Rio de Janeiro State and in the different cities were calculated per 100,000 inhabitants.
3. Analysis of sociodemographic characteristics: distribution of cases according to gender in different age groups, comparing the men/women variation (gender ratio) according to age progression. Missing data related to these variables were not considered in the analyses.
4. Georeferencing: it was performed using the coordinates (latitude and longitude) of the centroid of the cities of residence of the affected people. This georeferencing at the municipal level (cities) was performed for cluster analysis.
5. Clusters: refers to a geographic or spatiotemporal area where there is a statistically significant concentration of sporotrichosis cases regarding what would be expected by random distribution based on the size of the population and the territorial area. In this study, a proportion of 50% of the population was used to avoid negative clusters and the radius selected was 40 km. For these analyses, Space-Time scan statistics was used with the program SaTScan (version 10.1, July 2022, Boston, Massachusetts, USA). Poisson model was employed for the risk estimation (space-time) in the affected cities. This relative risk of the cluster considers the incidence rate of the cluster in relation to the incidence rate of the total affected territory. Construction of clusters was performed in the period of low incidence (2007-2015) and of high incidence (2016-2023). Thematic maps were elaborated to allow better visualization.

### Statistical analysis

Chi-square test was used to compare categorical variables and t-test to compare averages in two groups. Simple correlation analysis was used in numerical variables. Regression analysis was conducted for specific forecasting by determining the correlation (R), R^2^, adjusted R^2^ coefficients, and standard error of the estimate. Significance level was fixed at p < 0.05. Statistical analysis was conducted via R software (version 4.2.1, University of Auckland, Auckland, New Zealand).

## RESULTS

**Figure 1:**
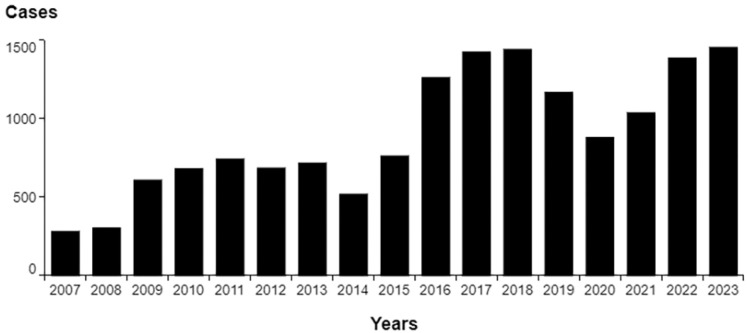
Distribution of human cases of Sporotrichosis in the Rio de Janeiro State, 2007-2023.

In the Rio de Janeiro State, 15,401 new cases of sporotrichosis were reported for 2007–2023. Our analyses show a positive correlation between sporotrichosis cases and the studied years (R = 0.839; R^2^ = 0.704; adjusted R^2^ = 0.384; p-value = 0.001; confidence interval [CI] = 0.007 ∼ 0.015). This indicates that the number of cases increases as the years go by. However, there is a greater increase from 2015 onwards (Figure 1).

Table 1 shows the distribution of cases in association with age groups and gender. Most cases (64.4%) occurred in women, with a ratio of 1.8:1. The ratio between women and men intensified with increasing age, with the women/man ratio exceeding two from the age of 40.

**Table 1:** Socio-demographic characteristics and outcome of patients with sporotrichosis in the Rio de Janeiro State, Brazil, 2007–2023

Characteristic		Woman		Man		Total	
		n	%	n	%	n	F/M*
Age range	0–9	444	4.5	436	7.9	880	1.0
	10–19	882	8.9	697	12.7	1,579	1.3
	20–29	1,085	10.9	751	13.7	1,836	1.4
	30–39	1,484	15.0	776	14.1	2,260	1.9
	40–49	1,911	19.3	893	16.3	2,804	2.1
	50–59	1,901	19.2	882	16.1	2,783	2.2
	60–69	1,387	14.0	693	12.6	2,080	2.0
	≥ 70	820	8.3	359	6.5	1,179	2.3
		n	%	n	%	n	%
Race/skin color	White (W)	3,782	38.1	1,847	33.7	5,629	36.5
	Black (B)	705	7.1	415	7.6	1,120	7.3
	Mixed ethnicity	2,895	29.2	1,778	32.4	4,673	30.3
	Asian	58	0.6	27	0.5	85	0.6
	Indigenous	24	0.2	11	0.2	35	0.2
	No information	2,450	24.7	1,409	25.7	3,859	25.1
Residence zone	Urban	9,796	98.8	5,385	98.1	15,181	98.6
	Rural	118	1.2	102	1.9	220	1.4
Diagnosis	Epidemiological	3,356	33.9	1,755	32.0	5,111	33.2
	Laboratory	6,091	61.4	3,445	62.8	9,536	61.9
	No information	467	4.7	287	5.2	754	4.9
Infection site**	Yes	6,099	61.5	3,328	60.7	9,427	61.2
	No	250	2.5	172	3.1	422	2.7
	Indeterminate	3,565	36.0	1,987	36.2	5,552	36.0
Work-related	Yes	181	1.8	132	2.4	313	2.0
	No	6,302	63.6	3,373	61.5	9,675	62.8
	No information	3,431	34.6	1,982	36.1	5,413	35.1
Outcome	Cure	5,674	57.2	2,967	54.1	8,641	56.1
	Death	8	0.1	4	0.1	12	0.1
	Death (other causes)	10	0.1	15	0.3	25	0.2
	No information	4,222	42.6	2,501	45.6	6,723	43.7

* woman/man ratio;

** regarding city of residence.

Most of the infected people were in their productive phase of life, between 20 and 59 years old. The mean age in the women's group (43.81 years) was higher than in the men's group (39.59 years) (p-value 0.001, CI −4.86 ∼ −3.56). This difference between the means did not change over time. Most patients lived in urban areas (98.6%) and there were no differences between genders regarding this issue. White people (36.5%) and those with Mixed ethnicity (30.3%) predominated. There was no information on race in 25% of cases. Table 1 shows that most infections occurred in the cities of residence and were not related to work. Laboratory tests were used in most cases for the confirmatory diagnosis. More than 99% of patients were cured, considering those who had information related to the outcome. When comparing deaths by gender and age, women (eight cases) had twice as many cases of death from sporotrichosis compared to men (four cases). Among the women who died, 62.5% were over 40 years of age, while among the men only 25% were in this age group.

Analyzing gender, we observed that the proportion of women declined mildly over time (R = 0.55; R^2^ = 0.305; adjusted R^2^ = 0.259; p-value < 0.001; confidence interval = 0.849–0.932). In the first period (2007–2015), women accounted for 66.8% of cases, and in the second period (2016–2023), they accounted for 62.8%.

### Incidence analysis

**Figure 2:**
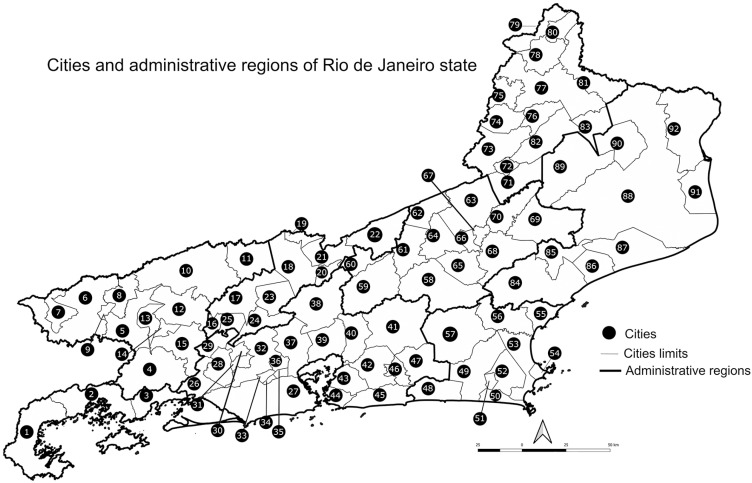
Cities of the Rio de Janeiro State numbered from center to right, according to Table 2.

The annual incidence was 5.6 cases per 100,000 inhabitants in the Rio de Janeiro State, from 2007 to 2023. Figure 2 shows the 92 cities in the state. Table 2 shows the cities grouped into their administrative regions, their populations, number of cases, and respective annual incidences of sporotrichosis for the studied period. Of the 92 cities, 82 (89.1%) had cases of sporotrichosis between 2007–2023. Considering the population at risk, which includes only people of the affected municipalities, the incidence reached 5.9/100,000 inhabitants. The Metropolitan region had the highest number of cases (12,955 cases, 84.1%). Of the nine cities with more than 300 cases (2, 27, 32, 35, 36, 37, 39, 43, 45 in Figure 2), eight are in the Metropolitan region. Those nine cities had 11,870 people with sporotrichosis in the studied period, which represented 77.1% of all reported cases. Regarding the administrative regions, the highest incidence rates were found in the Green Coast (11.1) and Metropolitan (6.3) regions, while the lowest incidence rates were found in the North (2.0) and Northwest (1.0) regions.

**Table 2:** Cities and administrative areas of the Rio de Janeiro State in terms of to their populations, and average incidence rate of sporotrichosis during 2007–2023 according to data from the Information System for Notifiable Diseases (SINAN).

Administrative region / City	Population	Cases	Incidence	Administrative region / City	Population	Cases	Incidence
GREEN COAST REGION	253,897	480	11.1	COASTAL LOWLANDS REGION	868,285	581	3.9
1. Paraty	45,243	105	15.1	48. Saquarema	89,559	78	5.6
2. Angra dos Reis	167,434	347	12.2	49. Araruama	129,671	115	5.5
3. Mangaratiba	41,243	28	4.1	50. Arraial do Cabo	30,986	31	5.9
**PARAIBA VALLEY REGION**	**865,130**	**559**	**3.8**	51. Iguaba Grande	27,920	16	3.7
4. Rio Claro	17,401	19	6.2	52. Sao Pedro da Aldeia	104,029	23	1.4
5. Barra Mansa	169,894	77	2.5	53. Cabo Frio	222,161	28	0.8
6. Resende	129,612	106	4.9	54. Armacao dos Buzios	40,006	21	3.9
7. Itatiaia	30,908	17	3.3	55. Rio das Ostras	15,491	251	11.4
8. Quatis	13,682	0	0.0	56. Casimiro de Abreu	17,198	8	1.2
9. Porto Real	20,373	10	3.2	57. Silva Jardim	21,352	10	2.7
10. Valenca	68,088	18	1.4	**MOUNTAINOUS REGION**	**522,500**	**208**	**2.3**
11. Rio das Flores	8,954	2	1.3	58. Nova Friburgo	189,939	71	2.2
12. Barra do Pirai	92,883	16	1.0	59. Teresopolis	165,123	100	3.4
13. Volta Redonda	261,563	277	6.1	60. S. Jose do Vale do Rio Preto	22,080	3	0.8
14. Pinheiral	24,298	10	2.4	61. Sumidouro	15,206	0	0.0
15. Pirai	27,474	7	1.5	62. Carmo	12,958	19	6.3
**CENTER-SOUTH REGION**	**278,628**	**247**	**5.2**	63. Cantagalo	8,741	2	0.6
16. Mendes	17,502	8	2.6	64. Duas Barras	10,980	0	0.0
17. Vassouras	33,976	40	6.6	65. Bom Jardim	28,102	6	1.3
18. Paraiba do Sul	42,063	30	4.1	66. Cordeiro	20,783	4	1.1
19. C. Levy Gasparian	46,110	1	0.5	67. Macuco	5,415	1	1.1
20. Areal	11,828	1	0.5	68. Trajano de Moraes	10,302	1	0.6
21. Tres Rios	78,346	107	7.9	69. Santa Maria Madalena	10,232	1	0.6
22. Sapucaia	17,729	4	1.3	70. Sao Sebastiao do Alto	7,750	0	0.0
23. Paty do Alferes	29,619	7	1.5	**NORTHWEST REGION**	**324,037**	**57**	**1.0**
24. Miguel Pereira	26,582	21	4.9	71. Itaocara	22,919	3	0.8
25. Eng. Paulo de Frontin	12,242	28	12.2	72. Aperibe	11,034	2	1.1
**METROPOLITAN REGION**	**12,021,871**	**12,955**	**6.3**	73. Santo Antonio de Padua	41,325	20	2.8
26. Itaguai	116,841	64	3.1	74. Miracema	26,881	0	0.0
27. Rio de Janeiro	6,211,223	6,642	6.0	75. Laje do Muriae	7,336	1	0.8
28. Seropedica	80,596	100	7.5	76. Sao Jose de Uba	7,070	1	0.8
29. Paracambi	41,375	48	5.8	77. Itaperuna	101,041	24	1.4
30. Japeri	96,289	164	9.6	78. Natividade	15,074	1	0.4
31. Queimados	140,523	201	8.2	79. Porciuncula	17,288	2	0.6
32. Nova Iguacu	785,867	1,587	11.6	80. Varre-Sai	10,207	0	0.0
33. Mesquita	167,127	230	7.8	81. Bom Jesus do Itabapoana	35,173	1	0.2
34. Nilopolis	146,774	140	5.2	82. Cambuci	14,616	0	0.0
35. Sao Joao de Meriti	440,962	419	5.3	83. Italva	14,073	2	0.8
36. Belford Roxo	483,087	413	4.9	**NORTH REGION**	**920,826**	**314**	**2.0**
37. Duque de Caxias	808,161	851	5.7	84. Macae	246,391	69	1.7
38. Petropolis	278,881	66	1.3	85. Conceicao de Macabu	21,104	0	0.0
39. Mage	228,127	470	11.7	86. Carapebus	13,847	0	0.0
40. Guapimirim	51,696	66	7.0	87. Quissama	22,393	5	1.3
41. Cachoeiras de Macacu	56,943	12	1.2	88. Campos dos Goytacazes	483,540	229	2.8
42. Itaborai	224,267	97	2.5	89. Sao Fidelis	45,059	4	0.6
43. Sao Goncalo	896,744	736	4.2	90. Cardoso Moreira	19,390	0	0.0
44. Niteroi	481,749	165	1.9	91. Sao Joao da Barra	36,573	5	0.8
45. Marica	197,277	405	15.9	92. S. Francisco do Itabapoana	38,961	2	0.3
46. Tangua	31,086	23	4.2				
47. Rio Bonito	56,276	56	5.7				

Incidence = average incidence rates of sporotrichosis per 100,000 inhabitants, 2007-2023; bold capital letters = administrative regions; numbers according to the map in Figure 2.

Our cohort can be divided into two periods, according to the magnitude of the endemic disease: the first, which runs from 2007 to 2015, has a lower number of notifications and an annual incidence rate of 3.6 cases per 100,000 inhabitants; and the second, which runs from 2016 to 2023, has a higher number of notifications and an annual incidence rate of 7.4 cases per 100,000 inhabitants (RR 2.05, p-value 0.0001; CI 1.771 ∼ 1.893).

**Figure 3:**
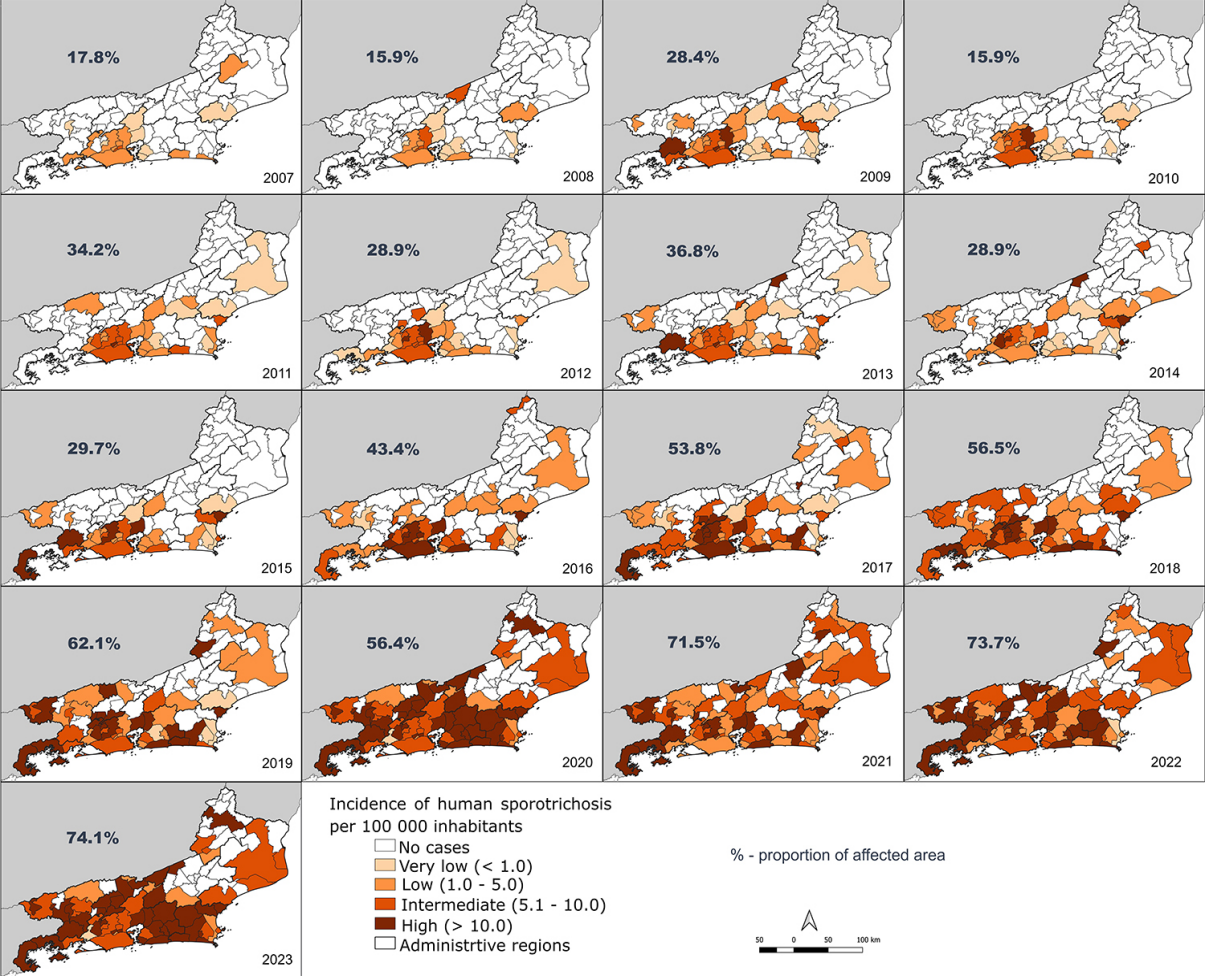
Incidence of sporotrichosis in the cities of the Rio de Janeiro State per year.

Figure 3 shows the annual incidence rate levels of sporotrichosis per 100,000 inhabitants in the different cities in each year of the period 2007–2023. Incidence rate levels corresponded to very low (< 1.0), low (1.0–5.0), intermediate (5.1–10.0), and high (> 10) incidence. The incidence rate levels have been increasing over time, as has the proportion of affected territories. Most cities have gone from very low to low incidences, from low to intermediate or high incidences. The area with cases, which represented an extension of 7,782.0 km^2^ (17.8%) of the state's territory in 2007, increased to 18,969.1 km^2^ (43.4%) in 2016, reaching 32,418.2 km^2^ (74.1%) of the territory in 2023. The very low incidence (< 1 case/100,000 inhabitants) and low incidence (1–5 cases/100,000 inhabitants), which represented an important portion of the endemic territory in the early years, corresponded to a much smaller portion of the endemic territory in recent years. Since 2020, a worsening of the endemic situation has been observed in the Rio de Janeiro State.

### Cluster analysis

**Figure 4:**
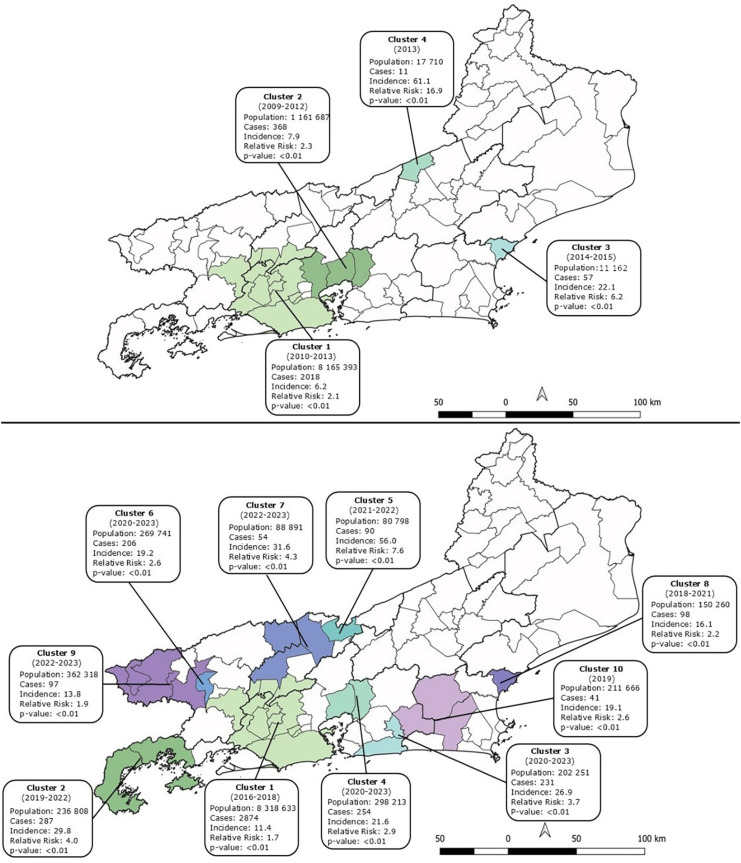
Clusters of human sporotrichosis in the Rio de Janeiro State in the periods: (A) 2007-2015; (B) 2016-2023.

The cluster analysis, considering space, incidence levels, and relative risk in the period, showed statistically significant clusters spread throughout Rio de Janeiro, in the periods of low incidence rate (2007-2015) and high incidence rate (2016-2023). Figure 4 shows these clusters with different heterogeneity indices. We can observe five elements comparing both periods: 1) There was a smaller number of clusters in the first period (four clusters) compared to the second period (ten clusters); 2) There was a greater number of cities incorporated into the clusters in the second period (32 cities), compared to the first period (18 cities); 3) The four clusters configured a smaller area (5,440.98 km^2^) in the low incidence rate period compared to the high incidence rate period (12,979.27 km^2^), which was equivalent to 2.38 times the affected territory in the first period. In the period of low incidence, the two main clusters were in the Metropolitan region and in some border cities. In the period of high incidence, the clusters had a greater range, being distributed in the Metropolitan, Green Coast, Paraiba Valley, Center-South, and Coastal Lowlands regions. The regions that did not show clusters in any period were the North and Northwest; 4) The relative risks of the clusters in both periods were heterogeneous, showing different probabilities of occurrence in the affected areas; 5) The clusters in this central area (Metropolitan region) had lower relative risks than the clusters in the more peripheral areas, both in the first period (2007–2015) and in the second period (2016–2023).

## DISCUSSION

Sporotrichosis has gained importance recently due to its prevalence in several countries around the world. The causative fungi have different ways of infection linked to human activities, lifestyles, and environmental and zoonotic interactions that influence their distribution and potential for geographic dispersion. In Brazil and some neighboring countries such as Argentina^22^ and Paraguay^23^, an increasing number of cases are mainly associated with zoonotic transmission through scratches or bites by infected cats. Rio de Janeiro has been considered the focus of this zoonotic endemic since the late 1990s, which has spread widely throughout the national territory^24^. Other Brazilian states were progressively affected in the Southeast^25^, South^26^, Northeast^27^, Central-West^28^, and North^29^ regions, where *S. brasiliensis* became the main agent responsible for transmission by cats.

In this study, we will discuss our results based on the peculiarities of Rio de Janeiro. Sporotrichosis is currently the fastest growing zoonosis in Rio de Janeiro, and if in the first studied years it covered less than 20% of the territory, recently it has affected more than 70% of the state. During the analyzed period, the expansion did not occur in a constant or homogeneous manner, nor did it have the same speed of cases over time, configuring different territorial risks related to epidemiological, cultural, geographic, and zoonotic nuances. This epidemiological dynamic is shown in the spatial analysis in the formation of significant clusters with different risks spread throughout the territory. These relative risks represent probabilities of territorial expansion. The clusters were more widespread in the second period, occupying previously unaffected areas.

One way to confirm this expanding trend was by analyzing the incidence rate levels. We concluded that the incidence of sporotrichosis, in all regions, without exception, increased over time, as it spread throughout the territory. This clear expansion did not show differences over time in age but showed a decline in the proportion of women.

Considering the number of cases and the high incidence level, the Metropolitan region (around the capital Rio de Janeiro city) seems to be the place from which the endemic disease spread to distant municipalities in the Rio de Janeiro State. Interestingly, this expansion occurred in a ‘hand fan-like’ fashion, in all directions. This epicenter presented lower relative risks in the clusters than the more peripheral areas, which shows that the probability of the occurrence of the disease intensifies as it expands. Theoretically, the incorporation of the disease into a new territory depends on the set of epidemiological, ecological, labor, cultural, and environmental characteristics. Thus, it seems that the south of the state (Green Coast and Paraiba Valley regions) has more favorable conditions for the expansion and endemic incorporation of the disease.

Although not all the variables related to this expansion are known, the role of the cat and the etiological agent are fundamental. Studies show that the feline epizootics precede the human endemics^24^. The movement of infected animals to areas free of the disease could allow the expansion of endemic areas^22^. However, this is not fully revealed. The behavior of a cat with access to the street enables the transmission between nearby cats, due to mating habits and fights over territorial disputes^30^. Nonetheless, molecular studies emphasize the existence of differences in susceptibility and potential for pathogenicity and virulence of different species in the *Sporothrix* genus, with *S. brasiliensis* being considered a particularly virulent species^13,15^.

Other findings were important in this study. There was a predominance of women, which did not change according to the geographic area or over time. Although there are no specific publications on sporotrichosis, this difference between genders could be explained by the greater contact with cats, since women are culturally more dedicated to caring for domestic animals. Therefore, there would be a greater probability of transmission to women if the cat became infected^31^. Since the beginning of the endemic disease due to the zoonotic transmission, in the late 1990s, the disease has occurred more in women who are not in the labor market or who conduct work activities in the domestic environment, predominantly in home care tasks, exposed to infection via contact/care/trauma with infected cats, which constitutes the profile of the most affected group^17^.

Studies conducted in other countries point to regional differences regarding gender and age, influenced mainly by the professional activities that favor transmission^32^. This is seen in rural workers in Colombia^33^ and Japan^6^, where the disease predominated in men. Women were more affected in Northeast China^5^. Note that there may be overlapping epidemiological patterns in the same country, due to sociocultural and labor characteristics. The states of Puebla^34^ and Guerrero^7^, in Mexico, had an inverted gender distribution: in the first, men predominated, and in the second, women.

In our study, most patients were in the productive phase of life. The age participation in the disease, like the gender variable, is linked to human activities with environmental and zoonotic interactions. In Peru, adults are the most affected in poor areas with a hot and humid climate^35^, whereas in other parts of the country there is a predominance among children^3^. There is a higher occurrence in adults in India^8^; in people over 50 years old in China^5^ and Japan^6^; and a balance between adults and children in Mexico^7,34^, where children in rural work were particularly affected^7^. Therefore, there seems to be no predisposition related to gender or a specific age group. Thus, in countries where sporotrichosis of sapronotic origin predominates, this diversity is influenced by the opportunity for exposure to the fungus via professional or recreational activities^36^. The disease was reported in Rio de Janeiro as being directly related to work in only 2% of cases, which is supported by the literature^17^.

Our data show that there was a predominance of White people, immediately followed by Black people and those with Mixed ethnicity (when aggregated). However, these data are not accurate, because they obey cultural factors of the understanding of the racial element. Nevertheless, there is much missing data regarding this variable. The literature points to similar results, with a predominance of White people^17^. This was observed at the time before the mandatory notification of cases and seems to have remained as a characteristic of the most affected group over time.

A very relevant result is the finding of 98.6% of the reported cases in individuals residing in urban areas. This profile of zoonotic sporotrichosis as a disease fundamentally of urban characteristics is corroborated by several studies conducted since the beginning of the endemic in the Rio de Janeiro State^17,32,37^, and clearly contrasts with the sapronotic cases in other areas of the world, fundamentally from rural areas^3,5,6,7,8,33^. A small percentage of cases in rural areas of Rio de Janeiro seems to be compatible with sporadic cases of sapronotic transmission that continue to occur amid zoonotic transmission. This hypothesis cannot be proven based on the notifications made to SINAN, due to the lack of information on the source of infection. However, during a short period (2019) in which the notification to SINAN in the Rio de Janeiro State was accompanied by the notification on a form that contained more detailed information about the mode of acquisition of the infection, contact with or handling of soil/garden was indicated as the exclusive source of infection in 11.7% of the cases confirmed by laboratory tests or clinical-epidemiological criteria^38^. It should be considered that infected cats, due to the abundance of fungi in their lesions, may also contaminate the soil and plants, therefore rendering them sources of infection^20,22^.

A total of 12 deaths attributed to sporotrichosis were detected over the 17 years of our study, but there is no description in the SINAN form regarding the cutaneous or disseminated form of sporotrichosis, nor information regarding comorbidities, such as immunosuppressive diseases, in these individuals. Literature has shown that deaths from sporotrichosis are often related to this class of diseases, particularly HIV infection^39,40^. Other factors, such as malnutrition, alcoholism, and unspecified immunodeficiencies, are also related to deaths from sporotrichosis^39^. Therefore, sporotrichosis should receive greater attention in these populations.

An important limitation of the study is the underreporting of cases, which hinders the epidemiological surveillance of sporotrichosis^26^ and has already been reported previously in a municipality in the Rio de Janeiro State^37^. Nevertheless, variables such as race/skin color, education, occupational exposure, and place where the infection occurred almost always showed relevant amounts of missing or inconsistent data in SINAN. Professionals should be made aware of the importance of these variables, which are fundamental to establishing effective control actions in vulnerable populations. Assessing the impact of poor reporting for some variables should be considered when adjusting the reporting system. We hope that the recent decision by the Brazilian Ministry of Health to make it a disease of mandatory notification in the national territory can improve the quality of notification, perhaps with the creation of a specific form for sporotrichosis in SINAN, facilitating the surveillance of the disease.

Understanding the dynamics of the disease and the epidemiological and ecological components of the endemic disease are essential for developing effective control programs. Human and feline sporotrichosis are treatable diseases. Rapid and accurate diagnosis is essential for the institution of antifungal treatment, which, in the case of cats, can interrupt the chain of transmission to humans and other animals. We must prevent the emergence of cases based on the diagnosis of infected human and cat cases, and on environmental studies. Ecological studies on reservoirs of *Sporothrix* spp. in the environment are necessary. Descriptive epidemiological analyses, complemented by spatial analyses, are important tools to support control measures to limit the expanding frontiers and emergence and re-emergence of sporotrichosis.

## CONCLUSIONS

The sporotrichosis endemics in the Rio de Janeiro State is dynamic and heterogeneous. Although it is expanding, there are temporal and spatial variations, configuring different risks. Urbanization processes, combined with cultural and zoonotic characteristics, favor the expansion of sporotrichosis. Contextualized research on affected territories is important to guide surveillance and control measures. Spatial analyses are essential for their potential to identify geographic patterns in the distribution of the disease, as they facilitate understanding of environmental, demographic, and social factors related to the worsening and spread of endemic diseases such as sporotrichosis.

## Data Availability

The complete anonymized dataset supporting the findings of this study is included within the article itself.
